# 5-Amino-3-(4*H*-1,2,4-triazol-4-yl)-1*H*-1,2,4-triazole

**DOI:** 10.1107/S1600536812034691

**Published:** 2012-08-11

**Authors:** Bing Liu, João P. C. Tomé, Luís Cunha-Silva, Filipe A. Almeida Paz

**Affiliations:** a Department of Chemistry, University of Aveiro, CICECO, 3810-193 Aveiro, Portugal; b REQUIMTE, Departamento de Química e Bioquímica, Faculdade de Ciências, Universidade do Porto, 4169-007 Porto, Portugal; c Department of Chemistry, University of Aveiro, QOPNA, 3810-193 Aveiro, Portugal

## Abstract

The asymmetric unit of the title compound, C_4_H_5_N_7_, comprises two independent but virtually superimposable mol­ecules. Each mol­ecule is planar with the dihedral angles between the five-membered rings being 2.8 (3) and 2.1 (3)°. The crystal structure is formed by an extensive network of relatively strong N—H⋯N hydrogen-bond inter­actions. Individual mol­ecules are arranged into supra­molecular zigzag chains running parallel to [001] by way of the strongest N—H⋯N inter­actions. Adjacent chains are inter­connected by rather long (*D*⋯*A* distances range from *ca* 3.00 to 3.03 Å) but highly directional (inter­action angles above *ca* 173°) hydrogen bonds forming a supra­molecular layer in the *bc* plane.

## Related literature

For the synthesis of 1-butyl-3-methyl­imidazolium bromide and 1,2-diformyl­hydrazine, see: Liu *et al.* (2007 ▶); Parnham & Morris (2006 ▶). For the use of triazole mol­ecules, see: Wang *et al.* (2012 ▶); Zhang *et al.* (2009 ▶). For previous research studies on crystal engineering approaches, see: Fernandes *et al.* (2011 ▶); Silva *et al.* (2011 ▶); Amarante *et al.* (2009 ▶); Paz & Klinowski (2007 ▶). For graph-set notation, see: Grell *et al.* (1999 ▶). For a description of the Cambridge Structural Database, see: Allen (2002 ▶).
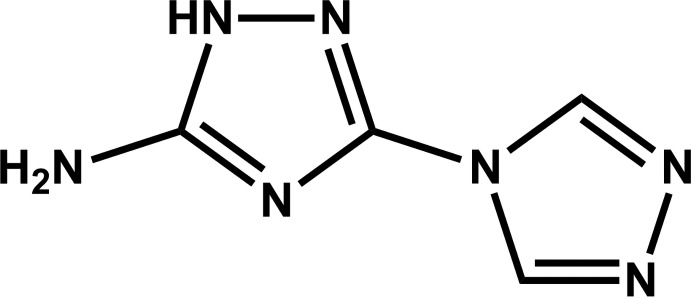


## Experimental

### 

#### Crystal data

C_4_H_5_N_7_
*M* *_r_* = 151.15
Monoclinic, 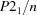

*a* = 13.765 (3) Å
*b* = 5.9378 (12) Å
*c* = 16.635 (4) Å
β = 112.914 (12)°
*V* = 1252.4 (5) Å^3^
*Z* = 8
Mo *K*α radiation
μ = 0.12 mm^−1^
*T* = 293 K
0.10 × 0.05 × 0.03 mm

#### Data collection

Bruker X8 Kappa CCD APEXII diffractometer
Absorption correction: multi-scan (*SADABS*; Sheldrick, 1998 ▶) *T*_min_ = 0.988, *T*_max_ = 0.996
8003 measured reflections
2192 independent reflections
975 reflections with *I* > 2σ(*I*)
*R*_int_ = 0.079

#### Refinement

*R*[*F* ^2^ > 2σ(*F* ^2^)] = 0.073
*wR*(*F* ^2^) = 0.226
*S* = 0.96
2192 reflections
217 parameters
8 restraints
H atoms treated by a mixture of independent and constrained refinement
Δρ_max_ = 0.52 e Å^−3^
Δρ_min_ = −0.36 e Å^−3^

### 

Data collection: *APEX2* (Bruker, 2006 ▶); cell refinement: *SAINT-Plus* (Bruker, 2005 ▶); data reduction: *SAINT-Plus*; program(s) used to solve structure: *SHELXTL* (Sheldrick, 2008 ▶); program(s) used to refine structure: *SHELXTL*; molecular graphics: *DIAMOND* (Brandenburg, 2009 ▶); software used to prepare material for publication: *SHELXTL*.

## Supplementary Material

Crystal structure: contains datablock(s) global, I. DOI: 10.1107/S1600536812034691/tk5136sup1.cif

Structure factors: contains datablock(s) I. DOI: 10.1107/S1600536812034691/tk5136Isup2.hkl

Supplementary material file. DOI: 10.1107/S1600536812034691/tk5136Isup3.cdx

Supplementary material file. DOI: 10.1107/S1600536812034691/tk5136Isup4.cml

Additional supplementary materials:  crystallographic information; 3D view; checkCIF report

## Figures and Tables

**Table 1:** Hydrogen-bond geometry (Å, °)

*D*—H⋯*A*	*D*—H	H⋯*A*	*D*⋯*A*	*D*—H⋯*A*
N1—H1*A*⋯N14^i^	0.90 (1)	2.43 (2)	3.239 (6)	150 (4)
N1—H1*B*⋯N4^ii^	0.90 (1)	2.11 (1)	3.002 (6)	173 (5)
N2—H2⋯N13^i^	0.90 (1)	1.93 (2)	2.772 (6)	155 (5)
N8—H8*A*⋯N7	0.90 (1)	2.43 (2)	3.264 (6)	154 (4)
N8—H8*B*⋯N11^iii^	0.90 (1)	2.14 (1)	3.034 (6)	176 (5)
N10—H10⋯N6	0.90 (1)	1.91 (2)	2.777 (6)	161 (5)

Symmetry codes: (i) 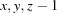; (ii) 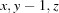; (iii) 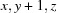.

**Fig. 1.**
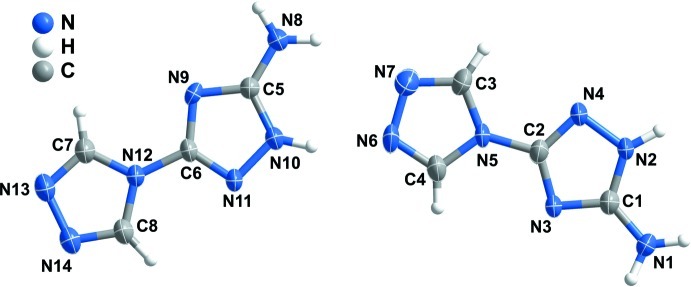
Asymmetric unit of the title compound showing all non-hydrogen atoms represented as anisotropic displacement ellipsoids drawn at the 50% probability level and hydrogen atoms as small spheres with arbitrary radius. The labeling scheme for all non-hydrogen atoms is also provided.

**Fig. 2.**
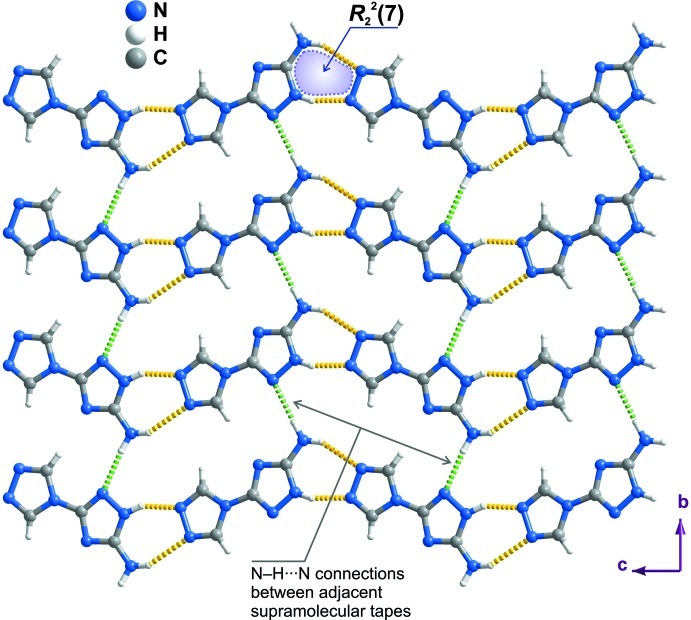
Two-dimensional supramolecular network placed in the bc plane formed by the N—H···N hydrogen bonding interactions between adjacent molecular units. Zigzag supramolecular tapes running parallel to the c axis of the unit cell are based on R22(7) graph set motifs (yellow dashed lines) formed between the 5-amino-3-(1,2,4-triazole) moiety of one molecule and the 1,2,4-triazole group from the adjacent one. Connections between adjacent tapes (dashed green lines) are ensured by additional N—H···N interactions along [010]. For geometrical details on the represented hydrogen bonding interactions see Table 1.

**Fig. 3.**
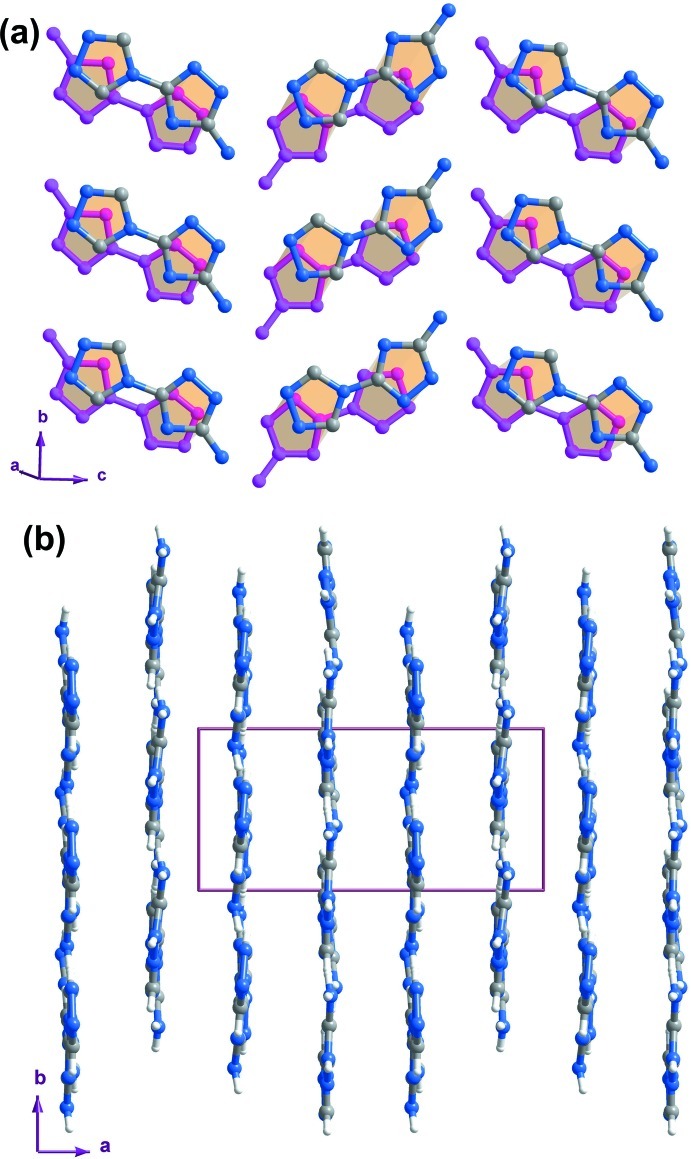
(a) Schematic representation of the supramolecular π-π interactions between HAtrtr molecules belonging to adjacent supramolecular layers. For clarity one layer is represented as pink bonds and all hydrogen atoms have omitted. For inter-centroid distances see Table 2. (b) Crystal packing of the title compound viewed along [001] direction, clearly showing the planar nature of the two-dimensional supramolecular layer.

## supplementary crystallographic information

### Comment

In the context of our interest in Crystal Engineering approaches (Wang *et al.* 2012; Fernandes *et al.*; 2011; Silva *et al.*, 2011; Amarante, Gonçalves *et al.*, 2009; Amarante *et al.*, 2009; Paz & Klinowski, 2007), we are currently designing new triazole molecules which could be simultaneously employed in the construction of novel Metal-Organic Frameworks (MOFs) or organic crystals. We note that this type of molecule has received considerable interest in the synthesis of polynuclear complexes and in the preparation of MOFs (Zhang *et al.*, 2009). A survey in the Cambridge structural Database (Allen, 2002) and in the literature revealed that the use of the asymmetrical bridging bitriazole molecule 5-amino-3-(1,2,4-triazol-4-yl)-1*H*-1,2,4-triazole (HAtrtr) has not been reported to date, either in MOFs nor in organic crystals. Furthermore, to the best of our knowledge, its crystal structure has not been reported. Following our recent efforts we were able to isolate good-quality single-crystals of the title compound as a minor secondary phase and here we wish to report its crystal structure at ambient temperature.

The asymmetric unit of the title compound (I) comprises two whole molecules (*i.e.*, *Z'*=2) of HAtrtr as depicted in Fig. 1. We note that these two individual moieties are almost perfectly overlaid by assuming a combination of both inversion and molecular flexibility (maximum distance of *ca* 0.021 Å with RMS of *ca* 0.014 Å). Nevertheless, this feature is not described by crystal symmetry as the "head-to-tail" orientation of the molecules can not be described by the screw-axis parallel to the *b*-axis of the unit cell. Both molecules are almost planar with the dihedral angles between the 1,2,4-triazole rings being only of *ca* 2.8 and 2.1°. In addition, the medium planes of all non-hydrogen atoms of each molecule subtend an angle of just *ca* 7.1°, indicating that the molecules can also be envisaged as coplanar.

HAtrtr is rich in groups capable of forming strong N—H···N hydrogen bonding interactions (see Table 1 for further geometrical details), which direct the crystal packing features of the title compound. The most striking intermolecular interactions concern the double donation of hydrogen atoms from each 5-amino-3-(1,2,4-triazole moiety to the 1,2,4-triazole group of an adjacent molecule (N1—H1A···N14, N2—H2···N13, N8—H8A···N7 and N10—H10···N6), forming a *R*_2_^2^(7) graph set motif (dashed yellow lines in Fig. 2) (Grell *et al.*, 1999). This supramolecular motif constitutes the basis of the formation of a supramolecular zigzag tape parallel to the *c* axis, for which the repeating motif are the two molecules composing the asymmetric unit. Tapes are interconnected by additional N—H···N interactions (N1—H1B···N4 and N8—H8B···N11) which, despite being slightly long (*d*_D···A_ ranging from 3.002 (6) to 3.034 (6) Å), are nevertheless highly directional with the interaction angles approaching linearity (of *ca* 173 and 176°). Noteworthy, these inter-chain connections are further strengthened by the presence of two weak C—H···N interactions (not represented): C3—H3···N3^i^ with *d*_C···N_=3.389 (6) Å and <(CHN)=174°; C8—H8···N9^ii^ with *d*_C···N_=3.435 (6) Å and <(CHN)=167° (symmetry codes: (i) *x*, 1 + *y*, *z*; (ii) *x*, -1 + *y*, *z*). The combination of these N—H···N and C—H···N contacts can be described by the *R*_2_^2^(8) graph set motif.

The hydrogen bonding interactions connecting adjacent molecular units and summarized in the previous paragraph lead to the formation of a two-dimensional supramolecular layer placed in the *bc* plane as shown in Fig. 2. Individual layers close pack parallel to the *a*-axis of the unit cell mediated by offset π-π contacts between HAtrtr molecules (Fig. 3 and Table 2 for intercentroid distances). Noteworthy, the inter-layer distance along the *a* axis alternates: layers are disposed into pairs so to promote the aforementioned strong offset π-π contacts within one pair; connections between adjacent pairs of supramolecular layers are weaker (*i.e.* longer inter-centroid distances - not shown).

### Experimental

3,5-Di(amino)-1,2,4-triazole (98% purity) and Mn(OAc)_2_^.^4H_2_O (99%+ purity) were purchased from Sigma-Aldrich and were used as received without further purification. 5-Amino-3-(1,2,4-triazol-4-yl)-1*H*-1,2,4-triazole and 1,2-diformylhydrazine were prepared by methods reported in the literature (Liu *et al.*, 2007). 1-Butyl-3-methylimidazolium bromide ([BMI]Br) was also prepared according to literature procedures (Parnham & Morris, 2006) (pale-yellow oil; yield: 78°).

1,2-Diformylhydrazine (0.1 mol; 8.8095 g) and 3,5-di(amino)-1,2,4-triazole (0.05 mol; 4.9564 g) were mixed in a 25 mL Teflon-lined stainless-steel reaction vessel and heated at 170 °C for 3 days in a furnace. After this time, the vessel was allowed to cool slowly to ambient temperature yielding 5-amino-3-(1,2,4-triazol-4-yl)-1*H*-1,2,4-triazole (HAtrtr) as a white microcrystalline powder. The solid was then washed with copious amounts of water and ethanol and dried at *ca* 60 °C for 24 h. Yield: 6.85 g, 67.4%.

HAtrtr (0.2 mmol, 0.0407 g) and Mn(OAc)_2_^.^4H_2_O (1.0 mmol, 0.2451 g) were mixed with *ca* 0.50 g of [BMI]Br in a 25 mL teflon-lined stainless-steel reaction vessel. The resulting mixture was heated to 110 °C for 7 days. The vessel was then allowed to cool to ambient temperature at a rate of *ca* 1 °C/h. Small colourless platelets of HAtrtr were formed as a minor secondary product, whose crystal structure is reported in this manuscript.

### Refinement

Hydrogen atoms bound to carbon were placed at their idealized positions with C—H = 0.93 Å and included in the final structural model in the riding-motion approximation with isotropic displacement parameters fixed at 1.2×*U*_eq_(*C*).

Hydrogen atoms associated with nitrogen have been directly located from difference Fourier maps and were included in the structural model with the N—H and H···H (only for the –NH_2_ moieties) distances restrained to 0.900 (5) and 1.560 (5) Å, respectively, in order to ensure a chemically reasonable environment. These hydrogen atoms were refined using a riding-motion approximation with isotropic displacement parameters fixed at 1.5×*U*_eq_(N).

### Crystal data

**Table:**

C_4_H_5_N_7_	*F*(000) = 624
*M**_r_* = 151.15	*D*_x_ = 1.603 Mg m^−^^3^
Monoclinic, *P*2_1_/*n*	Mo *K*α radiation, λ = 0.71073 Å
Hall symbol: -P 2yn	Cell parameters from 823 reflections
*a* = 13.765 (3) Å	θ = 2.7–21.7°
*b* = 5.9378 (12) Å	µ = 0.12 mm^−^^1^
*c* = 16.635 (4) Å	*T* = 293 K
β = 112.914 (12)°	Prism, yellow
*V* = 1252.4 (5) Å^3^	0.10 × 0.05 × 0.03 mm
*Z* = 8	

### Data collection

**Table:**

Bruker X8 Kappa CCD APEXII diffractometer	2192 independent reflections
Radiation source: fine-focus sealed tube	975 reflections with *I* > 2σ(*I*)
Graphite monochromator	*R*_int_ = 0.079
ω / φ scans	θ_max_ = 25.0°, θ_min_ = 3.7°
Absorption correction: multi-scan (*SADABS*; Sheldrick, 1998)	*h* = −16→16
*T*_min_ = 0.988, *T*_max_ = 0.996	*k* = −7→5
8003 measured reflections	*l* = −19→19

### Refinement

**Table:**

Refinement on *F*^2^	Primary atom site location: structure-invariant direct methods
Least-squares matrix: full	Secondary atom site location: difference Fourier map
*R*[*F*^2^ > 2σ(*F*^2^)] = 0.073	Hydrogen site location: inferred from neighbouring sites
*wR*(*F*^2^) = 0.226	H atoms treated by a mixture of independent and constrained refinement
*S* = 0.96	*w* = 1/[σ^2^(*F*_o_^2^) + (0.1189*P*)^2^] where *P* = (*F*_o_^2^ + 2*F*_c_^2^)/3
2192 reflections	(Δ/σ)_max_ < 0.001
217 parameters	Δρ_max_ = 0.52 e Å^−^^3^
8 restraints	Δρ_min_ = −0.36 e Å^−^^3^

### Special details

**Table:**

Geometry. All e.s.d.'s (except the e.s.d. in the dihedral angle between two l.s. planes) are estimated using the full covariance matrix. The cell e.s.d.'s are taken into account individually in the estimation of e.s.d.'s in distances, angles and torsion angles; correlations between e.s.d.'s in cell parameters are only used when they are defined by crystal symmetry. An approximate (isotropic) treatment of cell e.s.d.'s is used for estimating e.s.d.'s involving l.s. planes.
Refinement. Refinement of *F*^2^ against ALL reflections. The weighted *R*-factor *wR* and goodness of fit *S* are based on *F*^2^, conventional *R*-factors *R* are based on *F*, with *F* set to zero for negative *F*^2^. The threshold expression of *F*^2^ > σ(*F*^2^) is used only for calculating *R*-factors(gt) *etc*. and is not relevant to the choice of reflections for refinement. *R*-factors based on *F*^2^ are statistically about twice as large as those based on *F*, and *R*- factors based on ALL data will be even larger.

### Fractional atomic coordinates and isotropic or equivalent isotropic displacement parameters (Å^2^)

**Table:**

	*x*	*y*	*z*	*U*_iso_*/*U*_eq_	
N1	0.6176 (4)	−0.3457 (6)	−0.1686 (3)	0.0465 (13)	
H1B	0.625 (4)	−0.477 (4)	−0.140 (3)	0.070*	
H1A	0.625 (4)	−0.341 (7)	−0.2198 (16)	0.070*	
N2	0.6244 (3)	0.0473 (7)	−0.1487 (3)	0.0431 (12)	
H2	0.624 (4)	0.106 (9)	−0.1987 (18)	0.065*	
N4	0.6274 (3)	0.2015 (6)	−0.0855 (3)	0.0443 (12)	
C2	0.6268 (4)	0.0647 (8)	−0.0238 (3)	0.0346 (12)	
N3	0.6228 (3)	−0.1582 (6)	−0.0393 (2)	0.0326 (10)	
C1	0.6217 (4)	−0.1618 (8)	−0.1201 (3)	0.0368 (12)	
N6	0.6346 (3)	0.1685 (7)	0.1884 (3)	0.0477 (12)	
N7	0.6317 (3)	0.3877 (7)	0.1576 (3)	0.0493 (12)	
C3	0.6290 (4)	0.3720 (8)	0.0783 (3)	0.0449 (14)	
H3	0.6272	0.4936	0.0426	0.054*	
N5	0.6292 (3)	0.1502 (6)	0.0556 (2)	0.0339 (10)	
C4	0.6328 (4)	0.0325 (9)	0.1264 (3)	0.0420 (13)	
H4	0.6338	−0.1238	0.1302	0.050*	
N10	0.6263 (3)	0.2101 (6)	0.3519 (3)	0.0417 (12)	
H10	0.636 (4)	0.166 (9)	0.3037 (19)	0.063*	
N11	0.6323 (3)	0.0579 (6)	0.4167 (3)	0.0383 (11)	
C6	0.6227 (3)	0.1927 (7)	0.4747 (3)	0.0312 (11)	
N9	0.6124 (3)	0.4162 (6)	0.4575 (3)	0.0350 (10)	
C5	0.6144 (4)	0.4198 (8)	0.3774 (3)	0.0341 (12)	
N14	0.6344 (3)	−0.1263 (7)	0.6590 (3)	0.0441 (11)	
N13	0.6262 (3)	0.0938 (7)	0.6861 (3)	0.0487 (12)	
C7	0.6202 (4)	0.2269 (8)	0.6221 (3)	0.0440 (14)	
H7	0.6136	0.3827	0.6229	0.053*	
N12	0.6250 (3)	0.1088 (6)	0.5547 (2)	0.0342 (10)	
C8	0.6339 (4)	−0.1094 (8)	0.5814 (3)	0.0411 (13)	
H8	0.6390	−0.2311	0.5480	0.049*	
N8	0.6036 (4)	0.6025 (7)	0.3278 (3)	0.0509 (13)	
H8A	0.605 (4)	0.589 (7)	0.2746 (15)	0.076*	
H8B	0.615 (4)	0.737 (4)	0.355 (3)	0.076*	

### Atomic displacement parameters (Å^2^)

**Table:**

	*U*^11^	*U*^22^	*U*^33^	*U*^12^	*U*^13^	*U*^23^
N1	0.083 (3)	0.031 (2)	0.035 (3)	0.001 (2)	0.033 (3)	−0.005 (2)
N2	0.084 (3)	0.030 (2)	0.026 (3)	−0.003 (2)	0.032 (3)	−0.001 (2)
N4	0.083 (3)	0.033 (2)	0.028 (3)	−0.001 (2)	0.033 (2)	0.000 (2)
C2	0.045 (3)	0.036 (3)	0.026 (3)	0.004 (2)	0.017 (3)	−0.002 (2)
N3	0.051 (3)	0.030 (2)	0.022 (2)	0.0005 (17)	0.020 (2)	0.0000 (17)
C1	0.050 (3)	0.036 (3)	0.030 (3)	0.001 (2)	0.022 (3)	−0.001 (2)
N6	0.075 (3)	0.051 (3)	0.025 (3)	0.007 (2)	0.028 (2)	−0.002 (2)
N7	0.066 (3)	0.044 (3)	0.044 (3)	−0.008 (2)	0.028 (3)	−0.008 (2)
C3	0.067 (4)	0.042 (3)	0.030 (3)	−0.005 (3)	0.022 (3)	−0.011 (2)
N5	0.052 (3)	0.035 (2)	0.021 (3)	0.0012 (19)	0.022 (2)	−0.0004 (18)
C4	0.059 (4)	0.045 (3)	0.027 (3)	0.005 (2)	0.022 (3)	0.003 (3)
N10	0.073 (3)	0.032 (3)	0.029 (3)	0.004 (2)	0.029 (3)	0.004 (2)
N11	0.067 (3)	0.031 (2)	0.023 (2)	−0.0016 (19)	0.024 (2)	−0.0013 (19)
C6	0.043 (3)	0.033 (3)	0.022 (3)	0.000 (2)	0.017 (2)	0.002 (2)
N9	0.055 (3)	0.029 (2)	0.026 (2)	0.0003 (18)	0.021 (2)	−0.0082 (18)
C5	0.050 (3)	0.028 (3)	0.028 (3)	0.001 (2)	0.020 (3)	0.000 (2)
N14	0.061 (3)	0.045 (3)	0.030 (3)	0.002 (2)	0.022 (2)	0.006 (2)
N13	0.073 (3)	0.048 (3)	0.029 (3)	−0.002 (2)	0.024 (2)	0.003 (2)
C7	0.069 (4)	0.038 (3)	0.033 (3)	−0.001 (2)	0.029 (3)	0.000 (3)
N12	0.052 (3)	0.030 (2)	0.024 (3)	−0.0001 (18)	0.020 (2)	−0.0002 (18)
C8	0.059 (4)	0.039 (3)	0.032 (3)	0.003 (2)	0.026 (3)	0.004 (2)
N8	0.097 (4)	0.030 (2)	0.036 (3)	0.001 (2)	0.037 (3)	0.002 (2)

### Geometric parameters (Å, º)

**Table:**

N1—C1	1.345 (6)	N10—C5	1.346 (6)
N1—H1B	0.900 (5)	N10—N11	1.385 (5)
N1—H1A	0.899 (5)	N10—H10	0.901 (5)
N2—C1	1.335 (6)	N11—C6	1.299 (5)
N2—N4	1.382 (5)	C6—N9	1.353 (5)
N2—H2	0.899 (5)	C6—N12	1.410 (5)
N4—C2	1.312 (6)	N9—C5	1.344 (6)
C2—N3	1.346 (5)	C5—N8	1.336 (6)
C2—N5	1.403 (5)	N14—C8	1.293 (6)
N3—C1	1.337 (6)	N14—N13	1.401 (5)
N6—C4	1.303 (6)	N13—C7	1.302 (6)
N6—N7	1.394 (6)	C7—N12	1.346 (6)
N7—C3	1.307 (6)	C7—H7	0.9300
C3—N5	1.370 (6)	N12—C8	1.360 (6)
C3—H3	0.9300	C8—H8	0.9300
N5—C4	1.353 (6)	N8—H8A	0.899 (5)
C4—H4	0.9300	N8—H8B	0.900 (5)
			
C1—N1—H1B	114 (3)	C5—N10—N11	109.6 (4)
C1—N1—H1A	123 (3)	C5—N10—H10	129 (4)
H1B—N1—H1A	120 (4)	N11—N10—H10	121 (4)
C1—N2—N4	110.0 (4)	C6—N11—N10	100.6 (4)
C1—N2—H2	134 (4)	N11—C6—N9	118.7 (4)
N4—N2—H2	116 (4)	N11—C6—N12	120.8 (4)
C2—N4—N2	100.2 (4)	N9—C6—N12	120.5 (4)
N4—C2—N3	118.1 (4)	C5—N9—C6	100.6 (4)
N4—C2—N5	120.5 (4)	N8—C5—N9	125.8 (4)
N3—C2—N5	121.4 (4)	N8—C5—N10	123.7 (4)
C1—N3—C2	101.1 (4)	N9—C5—N10	110.5 (4)
N2—C1—N3	110.6 (4)	C8—N14—N13	106.2 (4)
N2—C1—N1	122.8 (5)	C7—N13—N14	106.9 (4)
N3—C1—N1	126.6 (5)	N13—C7—N12	110.9 (4)
C4—N6—N7	107.4 (4)	N13—C7—H7	124.5
C3—N7—N6	106.8 (4)	N12—C7—H7	124.5
N7—C3—N5	110.1 (5)	C7—N12—C8	104.6 (4)
N7—C3—H3	124.9	C7—N12—C6	127.8 (4)
N5—C3—H3	124.9	C8—N12—C6	127.6 (4)
C4—N5—C3	105.1 (4)	N14—C8—N12	111.4 (4)
C4—N5—C2	127.7 (4)	N14—C8—H8	124.3
C3—N5—C2	127.2 (4)	N12—C8—H8	124.3
N6—C4—N5	110.6 (4)	C5—N8—H8A	120 (3)
N6—C4—H4	124.7	C5—N8—H8B	117 (3)
N5—C4—H4	124.7	H8A—N8—H8B	120 (4)
			
C1—N2—N4—C2	0.4 (5)	C5—N10—N11—C6	0.3 (5)
N2—N4—C2—N3	−0.7 (6)	N10—N11—C6—N9	−0.9 (6)
N2—N4—C2—N5	−179.9 (4)	N10—N11—C6—N12	−179.6 (4)
N4—C2—N3—C1	0.7 (6)	N11—C6—N9—C5	1.0 (6)
N5—C2—N3—C1	180.0 (4)	N12—C6—N9—C5	179.8 (4)
N4—N2—C1—N3	0.0 (6)	C6—N9—C5—N8	177.7 (5)
N4—N2—C1—N1	179.4 (4)	C6—N9—C5—N10	−0.7 (5)
C2—N3—C1—N2	−0.4 (5)	N11—N10—C5—N8	−178.2 (5)
C2—N3—C1—N1	−179.8 (5)	N11—N10—C5—N9	0.3 (5)
C4—N6—N7—C3	−0.4 (5)	C8—N14—N13—C7	0.7 (5)
N6—N7—C3—N5	0.4 (6)	N14—N13—C7—N12	−0.6 (6)
N7—C3—N5—C4	−0.3 (5)	N13—C7—N12—C8	0.3 (6)
N7—C3—N5—C2	180.0 (4)	N13—C7—N12—C6	−178.9 (4)
N4—C2—N5—C4	−177.5 (5)	N11—C6—N12—C7	177.2 (4)
N3—C2—N5—C4	3.3 (7)	N9—C6—N12—C7	−1.5 (7)
N4—C2—N5—C3	2.1 (7)	N11—C6—N12—C8	−1.8 (7)
N3—C2—N5—C3	−177.1 (4)	N9—C6—N12—C8	179.4 (4)
N7—N6—C4—N5	0.2 (5)	N13—N14—C8—N12	−0.5 (5)
C3—N5—C4—N6	0.0 (5)	C7—N12—C8—N14	0.2 (5)
C2—N5—C4—N6	179.7 (4)	C6—N12—C8—N14	179.4 (4)

### Hydrogen-bond geometry (Å, º)

**Table:**

*D*—H···*A*	*D*—H	H···*A*	*D*···*A*	*D*—H···*A*
N1—H1*A*···N14^i^	0.90 (1)	2.43 (2)	3.239 (6)	150 (4)
N1—H1*B*···N4^ii^	0.90 (1)	2.11 (1)	3.002 (6)	173 (5)
N2—H2···N13^i^	0.90 (1)	1.93 (2)	2.772 (6)	155 (5)
N8—H8*A*···N7	0.90 (1)	2.43 (2)	3.264 (6)	154 (4)
N8—H8*B*···N11^iii^	0.90 (1)	2.14 (1)	3.034 (6)	176 (5)
N10—H10···N6	0.90 (1)	1.91 (2)	2.777 (6)	161 (5)

Symmetry codes: (i) *x*, *y*, *z*−1; (ii) *x*, *y*−1, *z*; (iii) *x*, *y*+1, *z*.

### Selected π-π contacts

**Table:**

π-π interaction	d(Cg···Cg)
Cg(1)···Cg(2)^i^	3.580 (3)
Cg(3)···Cg(4)^ii^	3.700 (3)

Symmetry codes: (i) 1-*x*, -*y*, -*z*; (ii) 1-*x*, -*y*, 1-*z*; Cg(1): centroid of the ring formed by N2, N3, N4, C1 and C2; Cg(2): centroid of the ring formed by N5, N6, N7, C3 and C4; Cg(3): centroid of the ring formed by N9, N10, N11, C5 and C6; Cg(4): centroid of the ring formed by N12, N13, N14, C7 and C8.
